# Symbiotic bacteria enable olive fly larvae to overcome host defences

**DOI:** 10.1098/rsos.150170

**Published:** 2015-07-29

**Authors:** Michael Ben-Yosef, Zohar Pasternak, Edouard Jurkevitch, Boaz Yuval

**Affiliations:** 1 Department of Entomology, The Hebrew University of Jerusalem, Rehovot 76100, Israel; 2 Department of Plant Pathology and Microbiology, The Robert H. Smith Faculty of Agriculture, Food and Environment, The Hebrew University of Jerusalem, Rehovot 76100, Israel

**Keywords:** symbiosis, Tephritidae, fruit phenology, olive fly, bacteria, secondary metabolites

## Abstract

Ripe fruit offer readily available nutrients for many animals, including fruit fly larvae (Diptera: Tephritidae) and their associated rot-inducing bacteria. Yet, during most of their ontogeny, fruit remain chemically defended and effectively suppress herbivores and pathogens by high levels of secondary metabolites. Olive flies (*Bactrocera oleae*) are uniquely able to develop in unripe olives. Unlike other frugivorous tephritids, the larvae maintain bacteria confined within their midgut caeca. We examined the interaction between larvae, their associated bacteria, and fruit chemical defence, hypothesizing that bacterial contribution to larval development is contingent on the phenology of fruit defensive chemistry. We demonstrate that larvae require their natural complement of bacteria (*Candidatus* Erwinia dacicola: Enterobacteriaceae) in order to develop in unripe olives. Conversely, when feeding on ripe fruit, larval development proceeds independently of these bacteria. Our experiments suggest that bacteria counteract the inhibitory effect of oleuropein—the principal phenolic glycoside in unripe olives. In light of these results, we suggest that the unique symbiosis in olive flies, compared with other frugivorous tephritids, is understood by considering the relationship between the fly, bacteria and fruit chemistry. When applied in an evolutionary context, this approach may also point out the forces which shaped symbioses across the Tephritidae.

## 1. Introduction

Fruits undergo extensive transformations during their ripening process, before maturing into a fleshy, energy-rich reward for seed dispersers [1,2]. Unripe fruit usually offer little nutrition and are resistant to attack by herbivores and pathogens due to high contents of secondary metabolites with anti-nutritive, antimicrobial, deterrent and toxic effects, ultimately securing a safe environment for the seeds to mature (e.g. [3,4]). The fate of secondary metabolites during the ripening process is usually to be neutralized or degraded [3–5]. Together with the build-up of fruit sugar content, decrease in acidity and softening of tissues which are generally observed during ripening, these processes eventually contribute to the high nutritional value of mature fruit [1]. The ecological consequence of this adaptive schedule for herbivores and microbes is that unripe fruit are usually off the menu.

Fruit flies (Tephritidae, subfamilies Dacinae and Trypetinae) form a large and diverse group of phytophagous species, many of which are frugivores that negatively affect fruit horticulture worldwide [6]. Commonly, flies associate with free-living, rot-inducing bacteria (mainly Enterobacteriaceae such as *Klebsiella*, *Pantoea* and *Enterobacter* spp.) which are inoculated into the fruit by ovipositing females and enhance larval nutrition with protein and essential nutrients [7–10]. Regardless of their affinity to the Enterobacteriaceae, most species of fruit flies show only a loose association with any particular bacterial species and, correspondingly, bacteria proliferate independently, outside the larval gut, at the expense of fruit tissues [10–12]. Additionally, most flies tend to avoid the inhibiting conditions in unripe fruit and preferentially lay their eggs in ripe fruit [13], which better support larval development [14–17], as well as bacterial proliferation [9,18,19].

The olive fly (*Bactrocera oleae*; Dacinae)—a notorious multivoltine, monophagous pest of olives (*Olea europaea*; Oleaceae)—represents a striking exception to this paradigm. Unlike other frugivorous Dacinae and Trypetinae, olive flies associate with a single, vertically transmitted bacterium (*Candidatus*Erwinia dacicola: Enterobacteriaceae; henceforth Erwinia dacicola), which is considered an obligate, co-evolved symbiont of the fly [20–23]. This bacterium is exclusively maintained within four large caeca of the larval midgut [11,21,24] and is unable to proliferate elsewhere, either in the fruit [21,25] or *in vitro* [20,21]. Other bacteria associated with olive flies (e.g. [21,26,27]) are usually found in small numbers and probably are transient residents in the gut [28].

Additionally, whereas most fruit flies develop in ripe fruit, olive flies predominantly use unripe olives, allowing them to complete several generations before ripe fruit become available [29,30]. This unique preference involves dealing with abundant secondary metabolites, the main of which is oleuropein—a bitter phenolic glycoside contributing up to 14% of the fruit's dry weight (e.g. [4,31,32]). Many phenolics are important components in the chemical arsenal of plants and act as strong protein alkylators once oxidized or deglucosylated, consequently inhibiting herbivores and pathogens [33–36]. Similarly, oleuropein forms highly reactive aldehyde and quinone agents when enzymatically activated (by plant β-glucosidase and phenoloxidase) [37,38]. In *Ligustrum* spp. (Oleaceae) activated oleuropein cross-links foliar proteins into high molecular weight aggregates while covalently binding their lysine amino residues [37]. Consequently, non-specialized insect herbivores feeding on *Ligustrum* suffer severe arrestment in growth due to reduced lysine content and nutritional value of dietary proteins [39,40]. Activated oleuropein additionally asserts a strong antimicrobial effect preventing bacteria and fungi from decomposing plant tissues [41,42]. Olives are considered to employ a similar defence mechanism [38,43,44]. However, the consequences of this defence response on the nutrition of olive fly larvae have never been directly demonstrated.

The unique attributes of the association between olive flies and bacteria compared with other fruit-infesting tephritids, considered with the unusual fruit chemistry of its preferred host, suggest a vital contribution of bacteria to the life cycle of this fly. Indeed, previous studies revealed that the use of antibiotics in the female diet or as a topical application to fruit prevented larval development in unripe olives [45–48]. Nevertheless, under these conditions, larval development was supported to some extent by ripe fruit [49]. Interestingly, the phenology of oleuropein during fruit ripening seems to correspond with these observations. Oleuropein accumulates to high levels during the first stages of fruit set and is gradually degraded during ontogeny, remaining low in mature, ripe fruit [4,31,32]. Concordantly, olives lose their characteristic bitterness and astringency.

In this study, we examined the interaction between olive fly larvae, their symbiotic bacteria and olive fruit chemistry. We hypothesized that unripe olives impose a major constraint on developing larvae by reducing the nutritional value of protein, and that larvae overcome this restriction by their symbiotic bacteria. Conversely, we posited ripe olives to offer a more relaxed nutritional environment in terms of protein nutritional quality, and accordingly that development will occur independently of the symbiotic microbiota. To test these hypotheses, we monitored the development of symbiotic and aposymbiotic larvae in unripe and ripe olives and examined the extent to which protein cross-linking took place and lysine was lost in these fruit.

## 2. Material and methods

### 2.1 Effect of bacteria and fruit phenology on larval development

We determined how bacteria and fruit maturity affect larval development by generating symbiotic and aposymbiotic larvae and monitoring their development in unripe (green) and ripe (black) olives. Manipulating the larval symbiotic microbiota was achieved by supplementing the female's diet with antibiotics, thus controlling the transmission of bacteria to their eggs. In these experiments, eggs were deposited in unripe and ripe fruit by wild females which developed in field-collected olives and ecdysed in the laboratory (full details regarding rearing, antibiotic treatment and generation of infested fruit are provided as electronic supplementary material).

Unripe and ripe fruit bearing the eggs of symbiotic and axenic females were weighed and incubated individually in transparent plastic cups sealed with a fine mesh. Developing larvae were extracted out of 5 to 10 olives sampled at 5, 8 and 11 days post-oviposition (larvae at the age of 3, 6 and 9 days, respectively; *n*=10–15 in each age group), anaesthetized in cold 95% ethanol and measured for body length (to the nearest 0.03 mm). Larvae were then preserved frozen (−20°C) in 95% ethanol for further analyses. Additionally, newly hatched, 1-day-old larvae generated from eggs incubated in saline (electronic supplementary material), as well as fully developed pre-pupal larvae leaving their fruit, were sampled, measured and preserved as described above.

The remaining olives (*n*=70–91 in each treatment group) were incubated until all larvae completed their development and pupated, or for a maximal period of 28 days. Mature larvae exiting their fruit to pupate were isolated into cotton plugged test tubes and their developmental period (days from egg to pupa) was recorded. Pupal weight was determined (to the nearest 0.01 mg) 5 days after pupation. At the end of the experimental period, all olives were dissected and checked for the presence of larvae or pupae. In all cases, only a few dead larvae were detected.

The experiment was replicated twice using olives of two cultivars collected from insecticide-free orchards in Korazim and Rehovot, Israel. Each replicate examined larval performance in the fruit of a single cultivar, picked from one tree. To ensure that none of the olives were naturally infested selected branches which bore premature fruit were enclosed by net early in season, thus preventing access to wild females. Picked fruit were visually inspected and verified to be free of oviposition punctures before use. Larval development was monitored in ‘Souri’ olives during October and December of 2011 (mean weight: 2.76±0.03 and 2.27±0.04 g, unripe and ripe fruit, respectively). ‘Manzanillo’ variety olives were used during December 2012 and February 2013 (mean weight: 3.88±0.05 and 4.17±0.05 g, unripe and ripe fruit, respectively).

Additionally, we examined the ability of mass-reared olive flies to develop in olives. Olive flies and other tephritids are routinely reared using synthetic larval diets for research purposes and mass-release of males in sterile insect technique control operations [28]. However, due to artificial rearing practices (e.g. use of antimicrobials for controlling diet contaminations), mass-reared flies usually acquire a markedly different microbiota than wild flies, containing very little if any of the naturally occurring symbiotic bacteria [22,28]. In the following experiments, mass-reared females oviposited in olives without being treated with antibiotics, allowing us to examine whether an altered bacterial microbiota can support larval development. Pupae of a hybrid cross-breed between females from a long-established ‘Demokritos’ laboratory colony (Crete, Greece) and wild males from Israel were kindly provided by the Biofly mass-rearing facility (Biobee, Israel). The ecdysed adults were maintained as described above except for adding antibiotics to their diet. Females coupled on the fourth day post-eclosion and oviposited in unripe or ripe ‘Souri’ olives picked in Kurazim during September and December 2011, respectively. Egg-bearing fruit were obtained and handled as described. Larval development was monitored by recording larval period and pupal weight.

### 2.2 Protein-binding and lysine-decreasing activities in olive fruit

We examined the ability of unripe and ripe ‘Souri’ olive extracts to cross-link proteins using previously described methods [37]. A protein of known molecular weight (ovalbumin) was incubated in unripe and ripe fruit extracts, allowing oleuropein to cross-link protein units together. The formation of ovalbumin aggregates was then visualized by separating the proteins in fruit extracts according to their size using sodium dodecylsulfate polyacrylamide gel electrophoresis (SDS-PAGE). Total amounts of amino acids in the resulting samples, as well as in non-treated ovalbumin were subsequently quantified by reverse phase high-performance liquid chromatography (HPLC). These analyses provided measures for the formation of high-molecular-weight protein aggregates and destruction of lysine in unripe and ripe fruit. Further details are provided in the electronic supplementary material.

### 2.3 Diversity analyses and quantification of gut bacteria

High-throughput sequencing was applied in order to analyse the gut bacterial community of symbiotic, third instar larvae developing in unripe and ripe ‘Souri’ olives (sampled 11 days post-oviposition, *n*=5 in each group), as well as of their corresponding ovipositing females (*n*=4–5 in each group). The gut microbiota of mass-reared females ovipositing in ripe ‘Souri’ olives (*n*=6) was additionally analysed. Analyses were performed using the Illumina MiSeq platform (Illumina, USA) and the 515F-806R primer pair [50].

Quantification of the bacterial population contained within the larval midgut caeca was achieved by direct bacterial counts. Symbiotic larvae extracted from unripe and ripe ‘Souri’ olives at the age of 6 and 9 days (*n*=20) were used in this analysis. Additionally, larvae of antibiotic-treated, wild females (9-day-old larvae, *n*=10), as well as larvae originating from mass-reared females (14- to 15-day-old larvae, *n*=9) were assayed. These larvae all developed in ripe ‘Souri’ olives. Further details regarding insect dissection, DNA extraction, sequencing data handling and bacteria quantification procedures are provided as electronic supplementary material.

## 3. Data analysis

### 3.1 Larval development

Regression analysis, performed on the mean values of larval body size in each age group, was used to examine the effects of ‘treatment’ (symbiotic or aposymbiotic), ‘larval age’ and ‘olive variety’ on larval development rate in either unripe or ripe olives.

To compare the length of symbiotic and aposymbiotic larvae of similar age groups, the effects of ‘treatment’ and ‘larval age’ were examined together with ‘olive variety’ as an additional factor by full factorial, three-way ANOVA. Analyses were performed separately for unripe and ripe fruit. Within the models, means were separated by *a priori* *t*-test comparisons. Larval length readings were log-transformed prior to analysis in order to homogenize variances among groups (examined with Levene's test).

### 3.2 Pupal weight and larval period

The amount of resources available for each larva (‘fruit weight/no. of larvae’) was found to significantly affect pupal weight and larval period (linear regression analysis, see Results). Accordingly, we considered this factor as a covariate in the following analyses:

(1) To determine the effect of ‘treatment’ on pupal weight of larvae developing in ripe olives a three-way full factorial ANOVA including ‘fruit weight/no. of larvae’ and ‘olive variety’ was used. A similar analysis was applied to pupae resulting from unripe olives. However, as no aposymbiotic larvae developed, only the effects of ‘fruit weight/no. of larvae’ and ‘olive variety’ were addressed.
(2) The effects of ‘treatment’, ‘fruit weight/no. of larvae’ and ‘olive variety’ on larval developmental period were determined by parametric survival analysis in a full factorial design. In these analyses, ‘time to pupation’ was regarded as an event.

To meet the assumptions of linearity log-transformed values of ‘fruit weight/no. of larvae’ were used in these analyses. Additionally, the data of pupae which did not ecdyse into adults (dead pupae, often distinguished by accelerated weight loss) were omitted. Male and female data were examined separately.

### 3.3 Bacterial counts

Bacterial counts significantly and similarly correlated with larval length in symbiotic larvae (regardless of fruit maturity stage) but not in aposymbiotic larvae (determined by regression analysis and ANOVA, see Results). Accordingly, the effects of ‘treatment’ (symbiotic or aposymbiotic) on gut bacterial population counts were examined together with ‘larval length’ as a covariate by full factorial ANOVA, after pooling the data of symbiotic larvae developing in unripe and ripe olives.

Bacterial counts obtained from larvae of mass-reared females were not included in this analysis, but were examined for their correlation with larval length (regression analysis) and compared with counts obtained from aposymbiotic, wild larvae (ANOVA). Linearity and homoscedasticity were achieved by using Box–Cox transformed data.

All data were analysed using the JMP v. 9 statistical package (SAS, Cary, NC, USA). Means and their standard errors are reported.

## 4. Results

### 4.1 Effect of bacteria and fruit phenology on larval development

**Figure 1.**
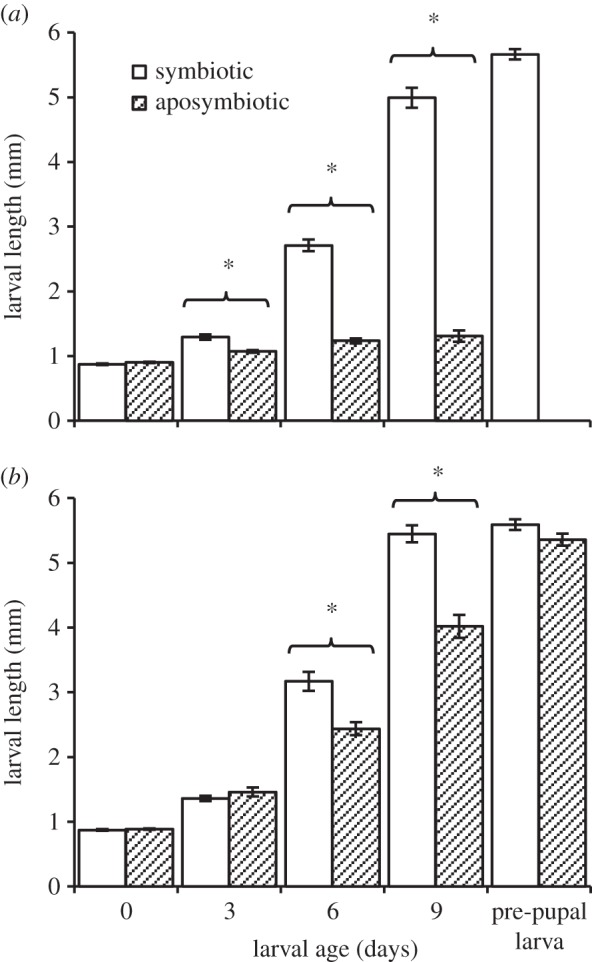
Mean body length of larvae developing in unripe (*a*) and ripe (*b*) olives, as affected by age and the presence (open bars) or absence (filled bars) of maternally transmitted symbiotic gut bacteria (symbiotic and aposymbiotic, respectively). Missing full bars correspond with failure of aposymbiotic larvae to survive. Asterisks represent significant differences among groups (*α*=0.05, *n*= 25–31 individuals in each group).

Consumption of antibiotics did not affect egg viability or size. Eggs laid by antibiotic-treated and non-treated females were equally viable (87.69±3.56% and 85.18±1.61% of eggs hatched into larvae, respectively). Additionally, newly hatched symbiotic and aposymbiotic larvae were similar in size (figure 1*a*,*b*).

Larvae significantly depended on their gut microbiota when developing in unripe olives (regression model specifications: *F*_7,8_=74.28, *p*<0.0001, *R*^2^=0.98). Examining the change in larval size according to age revealed a significant difference among the symbiotic and aposymbiotic groups (‘treatment’ × ‘larval age’ effect: *F*_1,8_=114.04, *p*<0.0001). This effect was common to both olive varieties (‘treatment’ × ‘larval age’ × ‘olive variety’ effect: *F*_1,8_=0.15, *p*=0.70). Accordingly, larval length was significantly affected by eliminating the gut microbiota (ANOVA model specifications: *F*_15,222_=188.51, *p*<0.0001, *R*^2^=0.92, figure 1*a*). Hatching as less than 1 mm long neonates, symbiotic larvae increased their length by nearly 6.5 times (from 0.87±0.01 mm to 5.66±0.08 mm) before completing development and exiting the fruit as pre-pupal larvae (figure 1*a*). Conversely, none of the aposymbiotic larvae developed into pre-pupal larvae or pupae. These larvae remained alive during the experimental period but never developed beyond the second instar and at 9 days old were only 1.4 times as long as newly hatched larvae (1.31±0.08 mm long, figure 1*a*). This arrestment in growth resulted in significant differences in body size between symbiotic and aposymbiotic 3-, 6- and 9-day-old larvae (ANOVA followed by *t*-test: *t*=3.84, *p*=0.0002; *t*=18.58, *p*<0.0001; *t*=32.42, *p*=<0.0001, respectively, figure 1*a*).

**Figure 2.**
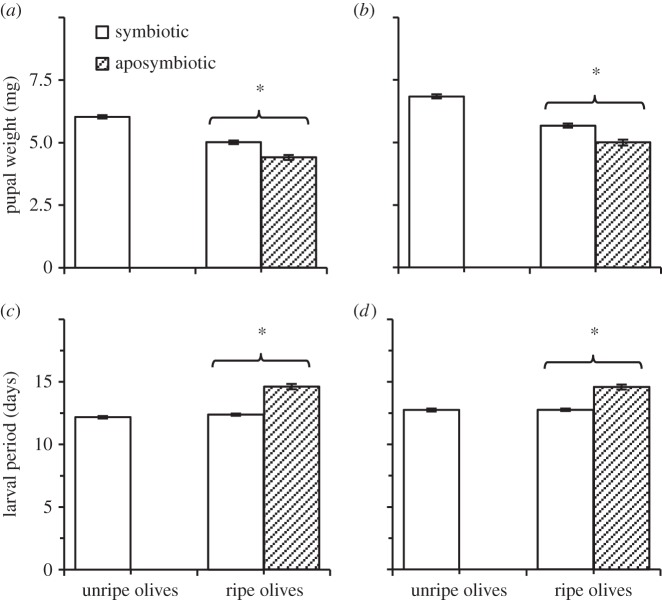
Mean pupal fresh weight and larval period of males (*a*,*c*) and females (*b*,*d*) developing in unripe and ripe olives, as affected by the presence (open bars) or absence (filled bars) of symbiotic gut bacteria (symbiotic and aposymbiotic larvae, respectively). Missing full bars correspond with failure of aposymbiotic larvae to survive. Asterisks represent significant differences among groups (*α*=0.05, *n*=89–175 individuals in each group).

The weight of male and female pupae resulting from symbiotic larvae (6.02±0.06 and 6.84±0.08 mg, figure 2*a*,*b*, respectively) was significantly affected by olive variety and the amount of resources available for each larva (ANOVA: *F*≥10.54, *p*<0.0001, *R*^2^≥0.16; for full model specifications, see electronic supplementary material, tables S1and S2). Larval period (12.18±0.09 and 12.75±0.11 days, males and females, figure 2*c*,*d*, respectively) was significantly affected by olive variety and in some groups by the amount of available resources (survival model specifications: *X*^2^≥62.12, d.f.=3, *p*<0.0001, see electronic supplementary material, tables S1 and S2).

Unlike unripe olives, ripe fruit supported the development of both symbiotic and aposymbiotic larvae (regression model specifications: *F*_7,8_=80.94, *p*<0.0001, *R*^2^=0.98). Nevertheless, aposymbiotic larvae developed significantly slower (‘treatment’ × ‘larval age’ effect: *F*_1,8_=7.05, *p*=0.02), regardless of olive variety (‘treatment’ × ‘larval age’ × ‘olive variety’ effect: *F*_1,8_=0.10, *p*=0.75). Comparing larval size at different time intervals (ANOVA model specifications: *F*_19,270_=385.44, *p*<0.0001, *R*^2^=0.96, figure 1*b*) showed that aposymbiotic larvae were significantly smaller than their symbiotic counterparts, 6 and 9 days after hatching (ANOVA followed by *t*-test: *t*=6.42, *p*<0.0001; *t*=8.75, *p*<0.0001, respectively, figure 1*b*). At the end of their development, aposymbiotic pre-pupal larvae achieved the same size as symbiotic larvae (5.35±0.09 and 5.58±0.08 mm, respectively, ANOVA followed by *t*-test: *t*=1.17, *p*=0.24, figure 1*b*). However, the weight of male and female pupae resulting from aposymbiotic larvae (ANOVA model specifications: *F*≥6.66, *p*<0.0001, *R*^2^=0.16) was significantly lower than that of their symbiotic counterparts (‘treatment’ effect: *F*≥26.5, *p*<0.0001)—an effect common to both olive varieties (electronic supplementary material, table S1). On average, these pupae were 11.99% lighter than symbiotic pupae (males: 4.40±0.09 and 5.01±0.07, figure 2*a*; females: 4.99±0.12 and 5.67±0.08, figure 2*b*; aposymbiotic and symbiotic insects, respectively). Additionally, olive variety and the amount of resources available for the larvae in each olive had a significant effect on pupal weight (see electronic supplementary material, tables S1 and S2).

The development of aposymbiotic male and female larvae (survival model specifications: *X*^2^≥152.36, d.f.=7, *p*<0.0001) was also associated with a significantly prolonged larval period compared with symbiotic larvae (‘treatment’ effect: *X*^2^≥44.58, d.f.=1, *p*<0.0001, electronic supplementary material, table S1, figure 2*c*,*d*, respectively), in both olive varieties (electronic supplementary material, table S1). On average, aposymbiotic larvae exited their fruit and pupated 2±0.2 days later than symbiotic larvae (males: 14.61±0.21 and 12.39±0.09 days, females: 14.58±0.19 and 12.77±0.98 days, respectively). Additional significant effects were also assigned to olive variety and the amount of resources available for the larvae in each olive (see electronic supplementary material, tables S1 and S2).

Mass-reared females readily oviposited in olives and egg viability was 89.9% and 100% in unripe and ripe fruit, respectively (based on 347 and 57 eggs examined). Larvae were not able to develop in unripe olives and none of the 50 unripe fruit in which females oviposited produced pupae. Examination of the olives 28 days post-oviposition revealed extensive tunnelling by the first instar larvae, indicating that newly hatched larvae actively fed but were not able to use the unripe fruit. Conversely, larvae were able to develop in ripe fruit, but were seemingly less successful than their wild counterparts. Out of the 61 egg-bearing, ripe fruit, 18 olives produced a total of 20 viable pupae with an average development time of 17.04±0.55 days (17.53±0.67 and 16±0.65 days; males and females, respectively), and average weight of 6.27±0.35 mg (6.08±0.26 and 6.74±0.20 mg; males and females, respectively).

### 4.2 Protein-binding and lysine-decreasing activities in olive fruit

**Figure 3.**
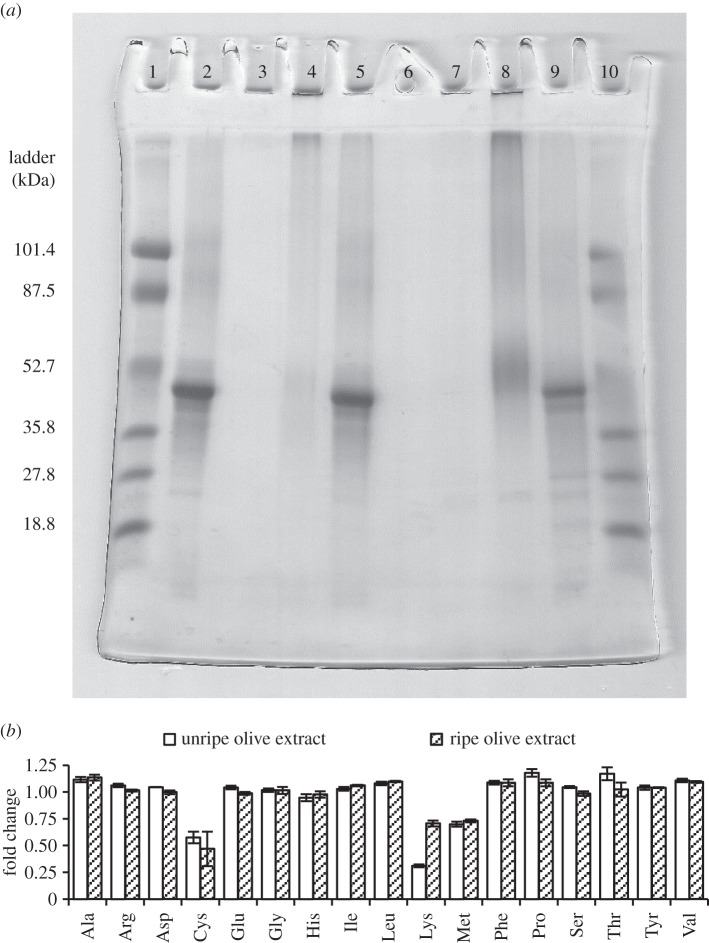
Protein cross-linking and lysine-decreasing activities in unripe and ripe ‘Souri’ olives. (*a*) SDS-PAGE analysis demonstrating the separation pattern of ovalbumin, either native (lane 2) or incubated in unripe (lane 4) or ripe (lane 8) olive extracts. Protein cross-linking associated with the unripe fruit (nearly no protein entered the gel) was reduced in ripe olives (substantial protein quantities visualized). In the presence of glycine, the modification of ovalbumin was prevented (lanes 5 and 9: unripe and ripe fruit, respectively). Crude extracts of unripe and ripe fruit (no ovalbumin or glycine added) contained very little protein (lanes 3 and 7, respectively). Lanes 1 and 10 were loaded with a protein size marker (‘low range’ standards; BioRad, USA). Lane 6 is empty. (*b*) Fold change in the amino acid content of ovalbumin (μmol mg^−1^ protein) after treatment in unripe (open bars) and ripe (shaded bars) ‘Souri’ olive extracts, as determined by HPLC quantitation (triplicate readings in each group). Lysine content was substantially reduced by unripe fruit extract.

SDS-PAGE was used to characterize the degree to which ovalbumin (figure 3*a*, lane 2) was cross-linked after treatment with unripe or ripe fruit extracts. Incubating ovalbumin in unripe olive extract resulted in the near complete disappearance of the band marking its expected position within the gel (figure 3*a*, lane 4). Instead, high molecular mass aggregates concentrated below the well of the stacking gel and in the upper part of the separating gel, suggesting that oleuropein was actively binding proteins in unripe fruit extract. In the presence of glycine (inhibitor of oleuropein [37]), ovalbumin remained free in solution and formed a visible band in the same position as untreated ovalbumin (figure 3*a*, lane 5), indicating that protein-binding activity was inhibited. Ripe olive extract had a reduced ability to bind proteins. When incubated in ripe fruit extract, some of the ovalbumin entered the separating gel forming a conspicuous smear, and concentrated approximately at the expected band position of untreated ovalbumin (figure 3*a*, lane 8). In the presence of glycine, the modification of ovalbumin by ripe fruit extract was inhibited (figure 3*a*, lane 9). Optical density analysis using the public domain NIH ImageJ software (http://imagej.nih.gov/ij/docs/guide/user-guide.pdf) indicated that lane 8 contained 81.79% of the protein present in lane 9 (ovalbumin in ripe fruit extract with or without glycine, respectively). Conversely, only 13.75% of the protein detected in lane 5 was present in lane 4 (ovalbumin in unripe fruit extract with or without glycine, respectively).

HPLC quantification of amino acids in ovalbumin treated with the extracts of unripe and ripe fruit revealed that the amounts of most amino acids remained relatively constant and were not affected by treatment (0.94–1.18 fold change compared with untreated ovalbumin, figure 3*b*). However, the quantities of lysine and, to a lesser extent, cysteine and methionine were reduced. Unripe olive extract had a pronounced effect on the lysine content of the protein, reducing it by 3.23 times compared with untreated ovalbumin (from 0.498 to 0.154 μmol mg^−1^ protein, figure 3*b*; electronic supplementary material, figure S1). The effect of ripe fruit extract was more subtle, and the detected lysine levels were only 1.38 times lower compared with untreated ovalbumin (0.350 and 0.498 μmol mg^−1^ protein, respectively, figure 3*b*; electronic supplementary material, figure S1). Cysteine was less prone to change by unripe fruit extract (1.73 and 2.28 fold reduction, unripe and ripe fruit extract, respectively), and methionine was similarly reduced in both treatment groups (1.43 and 1.38 fold reduction, unripe and ripe fruit extract, respectively, figure 3*b*).

### 4.3 Diversity analyses and quantification of gut bacteria

**Figure 4.**
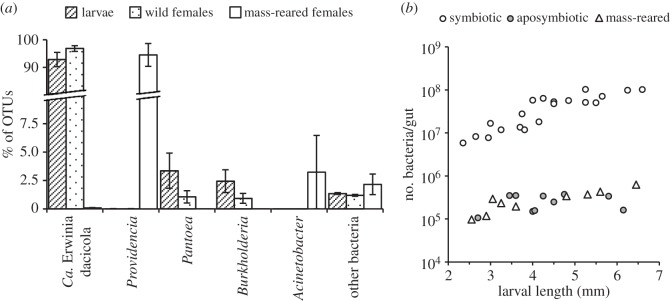
Diversity analysis and quantification of gut bacteria in olive flies. (*a*) High-throughput sequencing of the gut bacterial microbiome of third instar larvae developing in unripe (*n*=5) and ripe (*n*=5) olives, their female progenitors (*n*=5 and 4, respectively) and mass-reared females (*n*=6). Wild larvae and females had a homogeneous and constant microbiota consisting mainly of Erwinia dacicola. *Pantoea* and *Burkholderia* bacteria frequently co-occurred with Erwinia dacicola but were a minority. Mass-reared females had a significantly different microbiota consisting almost exclusively of *Providencia*bacteria. (*b*) Direct count quantification of bacteria in the midgut caeca of symbiotic larvae (*n*=20), aposymbiotic larvae (*n*=10) and larvae of mass-reared females (*n*=9) as affected by larval body size. Symbiotic larvae developed in unripe and ripe olives. Aposymbiotic and mass-reared larvae fed on ripe fruit.

High-throughput sequencing analysis of the gut bacterial community associated with adult wild females and their corresponding progeny developing in unripe and ripe ‘Souri’ olives identified an average of 5.4±3.4 operational taxonomic units (OTUs) per sample, after discounting OTUs which contained less than 10 reads. Rarefaction curves confirmed that all available diversity in the samples was detected (electronic supplementary material, figure S3). In all samples, three OTUs accounted for 98.17–99.24% of all obtained sequences (figure 4*a*). One of these—identified as Erwinia dacicola (more than 99% similarity to GeneBank accession no. HQ667589)—constituted, on average, 94.71±1.49% of all sequences in each sample. Depending on the abundance of Erwinia dacicola, two other OTUs identified as *Pantoea* sp. and *Burkholderia* sp. contributed the main part of the remaining sequences in these datasets (up to 20.9% of the total population). Other occurring OTUs were rare and together comprised 1.27±0.05% of the total population (figure 4*a*). Regardless of the above, the gut microbiotas of females and larvae were statistically similar, and independent of the fruit in which they oviposited or developed (MRPP tests, *A*<0.01, *p*>0.37).

The microbiotas of wild olive flies and mass-reared females were significantly different (MRPP test, *A*=0.87, *p*=0.00007). Mass-reared females harboured negligible levels of Erwinia dacicola in their gut, and associated almost exclusively with *Providencia* sp. bacteria (0.089±0.02 and 94.54±4.12% of all obtained sequences, respectively; figure 4*a*). One of the six females examined contained relatively low levels of *Providencia* sp. which were accompanied mainly by *Acinetobacter* sp. (73.92% and 19.4% of the sequences, respectively). Other bacteria (9.3±15.3 OTUs per sample) were rare and collectively constituted 2.15±0.9% of the gut population (figure 4*a*).

Sequencing data (prior to sub-sampling) were deposited at the MG-RAST repository (http://metagenomics.anl.gov/linkin.cgi?project=12936) under accession nos. 4622533.3–4622558.3 [51].

Direct counts of the bacteria contained within the midgut caeca of symbiotic larvae were significantly and positively correlated with larval length (regression analysis: *F*_1,8_>14.79, *p*<0.0049, *R*^2^>0.64), regardless of fruit maturity (ANOVA model specifications: *F*_3,16_=21.81, *p*=0.80, *R*^2^=0.80; ‘fruit maturity × larval length’ effect: *F*_1,16_=0.17, *p*=0.68) and did not differ among larvae developing in unripe and ripe fruit (‘fruit maturity’ effect: *F*_1,16_=0.80, *p*=0.38). Counts ranged between 5.79×10^6^ bacteria/gut in young, 6-day-old larvae and up to 102.25×10^6^ bacteria/gut in fully developed 9-day-old, third instar larvae (mean: 43.55±7.24×10^6^ bacteria/gut, figure 4*b*). However, second and third instar, 9-day-old larvae of antibiotic-treated females, which developed in ripe olives, harboured very little if any bacteria in their gastric caeca (mean: 0.25±0.03×10^6^ bacteria/larvae, figure 4*b*) regardless of their size (regression analysis: *F*_1,8_=0.38, *p*=0.55, *R*^2^=0.04), indicating that vertical transmission of bacteria was successfully terminated by administrating antibiotics to the females (ANOVA model specifications: *F*_3,26_=277.82, *p*<0.0001, *R*^2^=0.96; ‘treatment’ effect: *F*_1,26_=769.74, *p*<0.0001). Similarly, very few bacteria were detected in the gut of second and third instar larvae originating from mass-reared females, which developed in ripe olives (mean: 0.30±0.05×10^6^ bacteria/larvae, figure 4*b*). Here, bacteria counts correlated significantly with larval length (regression analysis: *F*_1,7_=27.16, *p*=0.0012, *R*^2^=0.79), but were statistically similar to counts of wild aposymbiotic larvae (ANOVA model specifications: *F*_3,15_=5.0, *p*<0.013, *R*^2^=0.50; ‘treatment’ effect: *F*_1,15_=0.55, *p*=0.46).

## 5. Discussion

### 5.1 Bacteria, fruit phenology and larval development

Diversity analysis of the bacterial population associated with wild adult females and their larvae revealed a homogeneous and constant microbiota consisting primarily of Erwinia dacicola and a small, varied consortium of other bacteria (figure 4*a*). These results together with previous findings [20–23] indicate that olive flies evolved to harbour, transmit and depend on one, obligate bacterial symbiont—Erwinia dacicola.

Erwinia dacicola was indispensable for the development of larvae in unripe, green olives. Treating females with antibiotics had no effect on egg viability and resulted in aposymbiotic larvae which actively fed during the experiments but failed to develop in unripe fruit (figures 4*b* and 1*a*). Conversely, symbiotic larvae successfully completed their development while accommodating a progressively growing population of bacteria in their midgut caeca. However, when feeding on ripe olives both symbiotic and aposymbiotic larvae completed their development (figure 1*a*,*b*). Thus, larvae strictly depend on their gut bacteria in order to develop in unripe olives but are able to mature independently of their microbiota in ripe fruit. Nevertheless, in ripe olives aposymbiotic larvae prolonged their feeding period by approximately 2 days and developed into pupae which weighed nearly 12% less than their symbiotic counterparts (figure 2). Hence, although not vital for development, Erwinia dacicola significantly accelerate development in ripe olives, probably by enhancing larval nutrition. These results indicate that Erwinia dacicola plays a vital role during larval development, enabling the utilization of unripe fruit.

As a gut resident, Erwinia dacicola is often accompanied by other bacteria (e.g. [21,26,27]) which are probably ingested with the diet and are transiently associated with the gut [28]. In our study *Pantoea* sp. bacteria were detected as relatively stable inhabitants in the gut. The abundance of these bacteria (together with *Burkholderia* sp.) seems to change correspondingly with the levels of Erwinia dacicola, suggesting a regulated interaction between these bacteria in the gut of olive fly larvae and adults (see also [21,24,25]).

In extreme situations, bacteria in the gut lumen and ceacae may be susceptible to replacement, e.g. during mass-rearing where the use of antimicrobials interrupts their natural transmission cycle and promotes the establishment of human-associated and environmental bacteria in the gut [28,52]. Indeed, in our experiments, mass-reared females had a significantly altered gut microbiota consisting mainly of non-native *Providencia* bacteria (figure 4*a*). The progeny of these females, similarly to aposymbiotic larvae, failed to develop in unripe olives but successfully reached adulthood when feeding on ripe fruit. The extremely reduced bacterial population in the gut of these larvae, regardless of the microbiota borne by their female progenitors, corresponds with this pattern and additionally suggests that bacteria other than Erwinia dacicola are poorly adapted to colonize the eggs or to propagate in the larval gut. The relatively poor performance of mass-reared larvae in ripe olives compared with wild, aposymbiotic larvae may result from genetic changes associated with domestication which promote the adaptation of flies to synthetic diets (e.g. [53]). Thus, although bacteria other than Erwinia dacicola may affect larval development, our experiments provide no evidence for this.

### 5.2 Protein availability and larval performance

The degree to which larvae depended on their bacteria is consistent with the extent to which lysine was lost and protein complexes accumulated following incubation of ovalbumin in fruit extracts. Notably, unripe olive extract had a greater capacity to bind ovalbumin units together and destroy lysine residues compared with ripe olive extract, suggesting that oleuropein—a potent protein cross-linker—is substantially more active in unripe olives (figure 3). Indeed, during early and mid-stages of fruit development oleuropein along with its direct precursor, ligstroside, and their derivatives remain highly abundant [4,32,54,55], and contribute up to 14% of fruit dry weight [31], and up to 94% of total fruit phenolics [32]. During ripening, the level of total phenols, including oleuropein, substantially declines and eventually reach low levels in ripe fruit [31,32,56]. Coupled with the decrease in phenol content, a ripening-associated reduction in fruit β-glucosidase—the main activating enzyme of oluropein—is observed [4,38,56], suggesting that the ability to activate oleuropein may also be impaired in ripe olives. Correspondingly, other studies show that the lysine content of olive pulp proteins tends to increase considerably during ripening, compared with other amino acids [57], indicating that the availability of this essential nutrient is subjected to substantial change during fruit ontogeny. Our results, supported by these findings indicate that the phenology of oleuropein is the main process determining the availability of lysine and total protein to feeding olive fly larvae. Thus, the accumulation of oleuropein during early fruit ontogeny, and its subsequent decline at later ripening stages is probably the main reason for the ripening-dependent ability of aposymbiotic larvae to use the fruit. Other ripening-associated processes which limit polyphenolics from interacting with proteins (e.g. increase in soluble pectic substances [5,58]) may further facilitate the development of aposymbiotic larvae in ripe fruit.

The ‘Souri’ and ‘Manzanillo’ olives, used in our experiments, are considered to be relatively abundant in polyphenolics (e.g. [59,60]). It would be interesting to examine larval dependency on bacteria in relation to lysine availability using fruits of other cultivars characterized by their low levels of polyphenolics [32].

In light of the above, we suggest that oleuropein is not necessarily toxic but acts indirectly as an anti-nutrient [34,37], imposing a major nutritional constraint on olive fly larvae by causing a severe deficiency in lysine—an essential nutrient [61]. Additionally, oleuropein may inactivate enzymes or reduce the digestibility of dietary protein [34,36,62], further impeding larvae from acquiring sufficient nutrients. Furthermore, any premature decomposition of the olive due to *in planta* bacterial proliferation, which may contribute to larval nutrition, is effectively prevented by activated oleorupein (e.g. [41]).Thus, when feeding on unripe fruit, larvae must contend with large, cross-linked protein aggregates which contain very little lysine and are probably recalcitrant to digestion. These restrictions are counteracted by the bacteria maintained in the midgut caeca. However, in ripe olives, where the impact of fruit defence becomes marginal and nutritional limitations are relaxed, larvae are able to tolerate the vestigial effects of oleuropein independently of their bacteria.

### 5.3 Contribution of bacteria to circumvention of host defence

Determining the precise mechanism by which the bacteria facilitate larval development in unripe olives is currently difficult, as Erwinia dacicola remains uncultivated and genomically uncharacterized. We assume that bacteria ultimately facilitate development by securing a source of digestible protein or amino acids for the larvae. This may be achieved by directly neutralizing plant defence chemicals as has been demonstrated for other insect-associated symbiotic fungi and bacteria [63–66], including bacteria associated with fruit flies [67,68]. Thus, secretion of polyphenol-degrading or resistant enzymes, polyphenol-binding polymers (e.g. [63,69]) or contribution of other counteracting agents (e.g. salivary amino acids; see electronic supplementary material, figure S2) may facilitate the dissociation of oleuropein–protein complexes in the gut and increase dietary protein digestibility. The phylogenetic proximity of this symbiont to necrotrophic free-living Erwiniae which exploit living plant tissues by secreting extracellular enzymes [70,71] supports this idea. In addition to their detoxification services, beneficial bacteria may also serve as a direct protein or amino acid source for the larvae—a contribution which we previously demonstrated to promote the fitness of adult olive flies [23,72]. The evolution of fruit flies from saprophagous ancestors which fed on microbe-rich, rotting plant tissues [73], and their unique physiological adaptations for lysing and digesting bacterial cells (as other cyclorhaphus dipterans) [74,75] lend some support to this notion. The contractile nature of the midgut caeca and the fact that bacteria are expelled into the gut during larval development [24] further suggest that bacteria are eventually digested. Thus, bacteria may additionally complement the larval diet by serving as a readily digestible and renewable source of balanced protein.

The effect of bacteria on larval development has scarcely been examined in other fruit flies and remains largely unknown. Direct examinations using artificial rearing media [76,77], together with a large body of indirect evidence (reviewed by Drew & Lloyd [7], Lauzon [8], Behar *et al*. [10] and Diaz-Fleischer *et al*. [73]) strongly suggest that bacteria contribute to larval development. However, in natural hosts, such effects may be vague and difficult to pinpoint [78]. Our results show that bacteria are less significant to larvae developing in ripe fruit (see also [78]). A similar scenario may take place in fruit cultivars having low levels of secondary metabolites [79]. Thus, the elusive role of bacteria during the larval stage may become apparent only in an ecologically relevant context, e.g. during a limited time frame in which fruit begin to ripen. During this period, bacterial proliferation may accelerate larval development, providing a significant evolutionary advantage, particularly in native hosts which retain high levels of chemical defence compared with domesticated cultivars [79].

### 5.4 Secondary plant metabolites and the promotion of host–symbiont co-speciation in fruit flies

Unlike olive flies, other frugivorous tephritids (subfamilies Dacinae and Trypetinae) which preferentially exploit ripe fruit do not rely on obligate, caecal symbionts but associate with rot-inducing bacteria and are severely inhibited in hosts where defence mechanisms are pronounced [14,79–81]. Thus, it seems that the magnitude of host defence and the mode by which larvae associate with bacteria in fruit-infesting tephritids are coupled, possibly by the ability of bacteria to proliferate in the fruit. Interestingly, olive flies share similar adaptations for housing and transmitting bacteria with members of the highly evolved Tephritinae subfamily, which predominantly reproduce in flower receptacles of the Asteraceae [12,82,83]. These flies are similarly associated with obligate, maternally transmitted, co-evolved bacteria (e.g. *Ca.* Stamerulla spp.), which occupy the larval midgut caeca and are phylogenetically related to Erwinia dacicola [70,84,85]. Such mutual traits shared by flies which are phylogenetically distant may have evolved independently as a result of similar constraints shaping the association with bacteria during the larval stage. Accordingly, an emergent principle from this and previous studies is that the intensity of plant defence exhibited by the hosts of tephritid flies determines the mode whereby larvae associate with bacteria. When considered together with the unique chemistry of the olive fruit, the dependence of the olive fly on one obligatory bacterial mutualist illustrates this principle. We assume that obligate symbioses evolved where host plants evince anti-nutritive and antimicrobial properties, inhibiting bacteria and larvae alike. Indeed, the defencive chemistry in the Asteraceae is largely dependent on sesquiterpene lactones—strong alkylating defence molecules—which act similarly to oleuropein (reviewed by Felton & Gatehouse [34] and Schmidt [86,87]), suggesting that the Tephritinae, similarly to olive flies, feed and develop in plant tissues rich in anti-nutritive and antimicrobial secondary metabolites. During the adaptation to such hosts bacteria which successfully colonized the protected lumen of the larval caeca were probably naturally selected. Subsequently, under the confined, stable conditions in the gut, a single bacterium suited to living within the insect host could have evolved. The importance of these bacteria for larval development probably perpetuated their transmission cycle and prevalence in the adult stage as well, eventually leading to close co-speciation. Conversely, in ripe fruit where most frugivorous tephritids develop, bacteria readily proliferate independently of the larvae at the expense of plant tissues. In such conditions, any particular bacterium is less prone to be selected. Indeed, a varied and commutative consortium of associated bacteria, responsive to host plant chemistry, may have promoted the ability of many of these flies to colonize different fruit (i.e. polyphagy). Accordingly, any selective pressure for maintaining a constant and obligate microbiota in larvae and adult flies would be more relaxed.

## 6. Conclusion

The evolution of tephritid flies advanced rapidly following a shift from saprophagy to phytophagy—an innovation which was probably mediated by the ability of females to inoculate environmental, rot-inducing bacteria into living plant tissues [73]. Most frugivorous flies (Dacinae and Trypetinae) follow this pattern and develop in decomposing material resembling the ancestral larval habitat [73]. Frugivorous olive flies, as well as the flower infesting Tephritinae, have further adapted to use plant tissues which do not support bacterial proliferation due to their high content of defensive chemicals. Their radiation into this trophic niche required the adoption and maintenance of specific bacteria in the midgut caeca—a mutualism which later led to obligate dependency and coevolution between these two parties.

## Supplementary Material

Supplementary methods and results are uploaded as online supporting information.

## Supplementary Material

Ben-Yosef et al. (RSOS-150170) - Supplementary results-revised.docx

## Data accessibility

Supplementary methods and results (files S1 and S2) are uploaded as electronic supplementary material. 16S rRNA read sequences from Illumina sequencing have been deposited at the MG-RAST digital repository (http://metagenomics.anl.gov/linkin.cgi?project=12936) under accession nos. 4622533.3–4622558.3 [51].
