# Supplementary material for: Symbiotic bacteria enable olive fly larvae to overcome host defences
Source: R Soc Open Sci. 2015 Jul 29;2(7):150170. doi: 10.1098/rsos.150170 (PMC4632588; doi:10.1098/rsos.150170)
Supplement: Supplementary methods and results are uploaded as online supporting information. [file rsos150170supp1.docx]

**Ben-Yosef et al. (RSOS 150170): Symbiotic bacteria enable olive fly larvae to overcome host defenses.**

**Supplementary methods**

**Effect of bacteria and fruit phenology on larval development:**

All experiments were conducted in a controlled environment (25±1.5°C, 65±10% RH and 16:8 light:dark cycle). Teneral, 1- 3 day old wild flies, which developed in field collected olives and ecdysed in the laboratory, were maintained on sucrose and water for 10-15 days. Females were subsequently segregated into two 5 liter cages (150 females per cage) for the next 10-12 days and supplied with a liquid diet consisting of 20% sucrose solution supplemented with yeast hydrolysate (10 mg / ml; Difco) as a source of amino-acids and vitamins. The diet of females in one cage was additionally supplemented with the antibiotic piperacillin (200 ug/ml), which was previously found to effectively suppress the gut microbiota of adult flies (1, 2). Diets were filter sterilized prior to use (0.2μm pore size filter; Whatman, Germany) and delivered through sterile capillary tubes which were replaced every 24 hours. On the 8th day of treatment 75 males of the same cohort were introduced into each cage to allow unmated females to copulate. Following the treatment period unripe or ripe olives were introduced into each cage where females concurrently oviposited in the fruit. Egg-bearing fruits were handled as described in the methods section of this paper.

Egg viability in the two treatment groups was estimated by allowing females to oviposit in artificial fruit (paraffin domes; 3) for the next 24 hours. The deposited eggs (n = 70 - 354, average: 229 per group) were subsequently collected and incubated in sterile saline (0.9% NaCl) for 72 hours, after which the newly hatched larvae and remaining unhatched eggs were counted to assess the proportional viability in each treatment group. Newly hatched larvae were measured for body length as described in the methods section of this paper.

**Protein binding and lysine decreasing activities in olive fruit:** protein cross-linking was examined in unripe and ripe 'Suri' olive using a previously described method (4, 5). Forty grams of freshly picked, uninfested fruit were chilled (4°C), destoned and homogenized in 76 ml of ice cold deionized distilled water (DDW) at 20000 RPM for 2 minutes, using a commercial blender. Particulate cell material and the lipid fraction were subsequently separated from the aqueous extract by centrifugation (10000 RCF for 10 minutes at 4°C). Assays were performed in triplicates in 1.5ml microfuge tubes containing 425µl of the resulting supernatant, 25µl of 20% ovalbumin solution in DDW and 50µl of 1M sodium phosphate buffer (pH 5.6 – 5.8) with or without 10% glycine - an inhibitor of oleuropein activity (6), (final concentrations: 1% ovalbumin and 1% or no glycine in 500µl of 0.1M sodium phosphate buffered fruit homogenate). Additionally, pure extracts (without ovalbumin or glycine) were included in these tests. Tubes were incubated open to allow oxygen in, at 25ºC for 2h with vigorous agitation. A 50µl sample of each solution was subsequently applied to sodium-dodecyl-sulfate polyacrylamide gel electrophoresis (SDS-PAGE) analysis in order to determine the degree to which ovalbumin was cross-linked fruit extracts. Proteins were separated in a 7.5-17.5% acrylamide gradient, using the Hoefer SE260 electrophoresis unit (Hoefer, USA), and visualized with coomassie-based commercial stain ('Imperial^TM^', Pierce, USA) according to the manufacturer's instructions. The remainder of each reaction solution was lyophilized in order to determine its dry weight. Total amounts of amino-acids in the resulting samples, as well as in non-treated ovalbumin were subsequently quantified by reverse phase high-performance liquid chromatography (HPLC) using the Waters Pico-Tag system (Waters, USA) at the Advanced Protein Technology Center of the Hospital for Sick Children, Ontario, Canada. The amino-acid composition of extract-treated ovalbumin was calculated by subtracting the quantities of amino-acids detected in pure fruit extracts from their correspondent amounts in extracts containing ovalbumin. Samples containing glycine were not analyzed.

**Diversity analyses and quantification of the gut microbiota:** Larvae and adult flies dedicated for these analyses were preserved frozen (-20°C) in 95% ethanol until processed. Insect dissection and DNA extraction procedures were performed in a sterile laminar flow hood. Prior to DNA extraction insects were externally rinsed in a mild detergent solution (1% Alconox, USA) for 1 minute and subsequently washed in sterile saline and sterile DDW. Larvae were dissected under a stereoscope using a pair of sterile forceps to extract the gastric caeca at the proximal section of the midgut. Similarly, the midgut and esophageal bulb of adult flies were extracted. Bacterial DNA was purified from the gut of each individual using the Chemagic DNA bacteria kit (Chemagen, Germany) according to the manufacturer's instructions. The 16S rRNA gene diversity in each sample was subsequently analyzed at the DNA Services Facility of the University of Illinois, Chicago, USA using the Illumina MiSeq platform (Illumina, USA) and the 515F-806R primer pair (7) targeting the V4 region of the bacterial 16S rRNA gene. Libraries from the 25 samples were constructed as previously described (8). Sequencing depth prior to subsampling was 73868±8677 reads per sample.

Obtained 16S rRNA sequences were processed and analyzed in MOTHUR v1.31 (9) as outlined (10) in the standard MOTHUR MiSeq protocol (http://www.mothur.org/wiki/MiSeq_SOP). Processing included forward and reverse read merging, quality trimming and exclusion of chimeras. To homogenize sample size, all samples were sub-sampled to include 50,000 sequences per sample, with an average read length of 250 bp. Sequences sharing 98% identity were clustered into the same operational taxonomic unit (OTU) and the representative sequence from each OTU was phylotyped based on release 9 of the RDP taxonomy (http://www.mothur.org/wiki/RDP_reference_files) with a bootstrap cutoff of 80. Coverage values exceeded 0.99 for all groups.

Multivariate analysis was performed in PC-ORD v6.08 (MjM Software, USA) with Sorensen distances. Ordinations were performed with non-metric multidimensional scaling (NMDS; 11) at 500 iterations, and cluster analyses were performed with flexible beta linkages (β = -0.25). Groups were statistically compared by multi-response permutation procedure (MRPP) tests (12).

**Quantification of gut bacteria:** Larvae intended for bacteria quantification assays were externally rinsed and dissected as described above, and the four midgut cecae were extracted and homogenized in 25µl (aposymbiotic or laboratory-reared larvae) or 25 - 200μl of sterile DDW (symbiotic larvae, depending on larval length). A sample of the bacterial suspension was loaded into a Petroff-Hausser counting chamber (Assitent, Germany) and the number of bacteria per gut was determined microscopically by directly counting the suspended cells. Sixteen fields were counted for each sample and counts were later averaged in order to determine the bacterial titer in the midgut caeca of each larva.

Contaminating bacteria (e.g. adhering to the integument) were detected by washing each larva in 25μl of sterile DDW prior to extracting the gut. The wash water was similarly examined for the presence of bacteria, in case of which the sample was regarded as contaminated and discarded.

**References**

1. Ben-Yosef, M., Jurkevitch, E., Yuval, B. 2008 Effect of bacteria on nutritional status and reproductive success of the Mediterranean fruit fly *Ceratitis capitata*. *Physiological Entomology,* **33,** 145-154.
2. Ben-Yosef, M., Aharon, Y., Jurkevitch, E., Yuval, B. 2010 Give us the tools and we will do the job: symbiotic bacteria affect olive fly fitness in a diet-dependent fashion. *Proceedings of the Royal Society B: Biological Sciences,* **277,** 1545-1552.
3. Hagen, K.S., Santas, L., Tsecouras, A. 1963 A technique of culturing the olive fly, *Dacus oleae* Gmel., on synthetic media under xenic conditions. *Radiation and Radioisotopes Applied to Insects of Agricultural Importance.* International Atomic Agency, Vienna, Athens, Greece.
4. Konno, K., Hirayama, C., Shinbo, H. 1997 Glycine in digestive juice: a strategy of herbivorous insects against chemical defense of host plants. *Journal of insect physiology,* **43,** 217-224.
5. Konno, K., Hirayama, C., Yasui, H., Nakamura, M. 1999 Enzymatic activation of oleuropein: a protein crosslinker used as a chemical defense in the privet tree. *Proceedings of the National Academy of Sciences,* **96,** 9159-9164.
6. Konno, K., Hirayama, C., Yasui, H., Okada, S., Sugimura, M., Yukuhiro, F., Tamura, Y., Hattori, M., Shinbo, H., Nakamura, M. 2010 GABA, β-alanine and glycine in the digestive juice of privet-specialist insects: convergent adaptive traits against plant iridoids. *Journal of chemical ecology,* **36,** 983-991.
7. Caporaso, J.G., Lauber, C.L., Walters, W.A., Berg-Lyons, D., Huntley, J., Fierer, N., Owens, S.M., Betley, J., Fraser, L., Bauer, M., Gormley, N., Gilbert, J.A., Smith, G., Knight, R. 2012 Ultra-high-throughput microbial community analysis on the Illumina HiSeq and MiSeq platforms. *ISME J,* **6,** 1621-1624.
8. Green, S.J., Venkatramanan, R., Naqib, A. 2015 Deconstructing the Polymerase Chain Reaction: Understanding and correcting bias associated with primer gegeneracies and primer-template mismatches. PLoS ONE **10**, e0128122.
9. Schloss, P.D., Westcott, S.L., Ryabin, T., Hall, J.R., Hartmann, M., Hollister, E.B., Lesniewski, R.A., Oakley, B.B., Parks, D.H., Robinson, C.J. 2009 Introducing mothur: open-source, platform-independent, community-supported software for describing and comparing microbial communities. *Applied and environmental microbiology,* **75,** 7537-7541.
10. Kozich, J.J., Westcott, S.L., Baxter, N.T., Highlander, S.K., Schloss, P.D. 2013 Development of a dual-index sequencing strategy and curation pipeline for analyzing amplicon sequence data on the MiSeq Illumina sequencing platform. *Applied and environmental microbiology,* **79,** 5112-5120.
11. Mather, P.M., Mather, P.M. 1976 *Computational methods of multivariate analysis in physical geography*. Wiley London.
12. Mielke Jr, P.W. 1984 Meteorological applications of permutation techniques based on distance functions. *Handbook of statistics* (eds P.R. Krishnaiah & P.K. Sen), pp. 813-830. Elsevier Science Publishers, Amsterdam.
