# Supplementary material for: Symbiotic bacteria enable olive fly larvae to overcome host defences
Source: R Soc Open Sci. 2015 Jul 29;2(7):150170. doi: 10.1098/rsos.150170 (PMC4632588; doi:10.1098/rsos.150170)
Supplement: Ben-Yosef et al. (RSOS-150170) - Supplementary results-revised.docx [file rsos150170supp2.docx]

**Ben-Yosef et al. (RSOS 150170): Symbiotic bacteria enable olive fly larvae to overcome host defenses.**

**Supplementary results: figures S1 – S3, tables S1 and S2**

**Figure S1:** High-performance liquid chromatography quantitation of amino acids in ovalbumin before (empty bars) and after treatment with unripe (full bars) or ripe (shaded bars) fruit extracts (triplicate readings in each group). Lysine was substantially reduced by unripe fruit extract.

**Figure S2:** During our experiments third instar aposymbiotic larvae developing in ripe olives often accumulated characteristic black deposits on the anterior part of their body ('head'), (a, b). Young aposymbiotic larvae, as well as mass-reared larvae similarly appeared with a black head. This condition was never observed in symbiotic larvae developing either in ripe (c, d) or unripe (e, f) fruit. This 'black-head syndrome' probably results from deposition of oxidized, polymerized phenolics during feeding, and suggests that salivary agents (e.g glycine and other amino-acids; see reference 39) may be involved in the inactivation of phenolics. If indeed this is the case for olive flies, bacteria may promote the secretion of such substances e.g. by indirectly fueling the nitrogen reserves of the larvae, allowing them to allocate nitrogen (amino-acids) for excretion in the saliva. The figure depicts the typical appearance of larvae developing in 'Suri' olives.


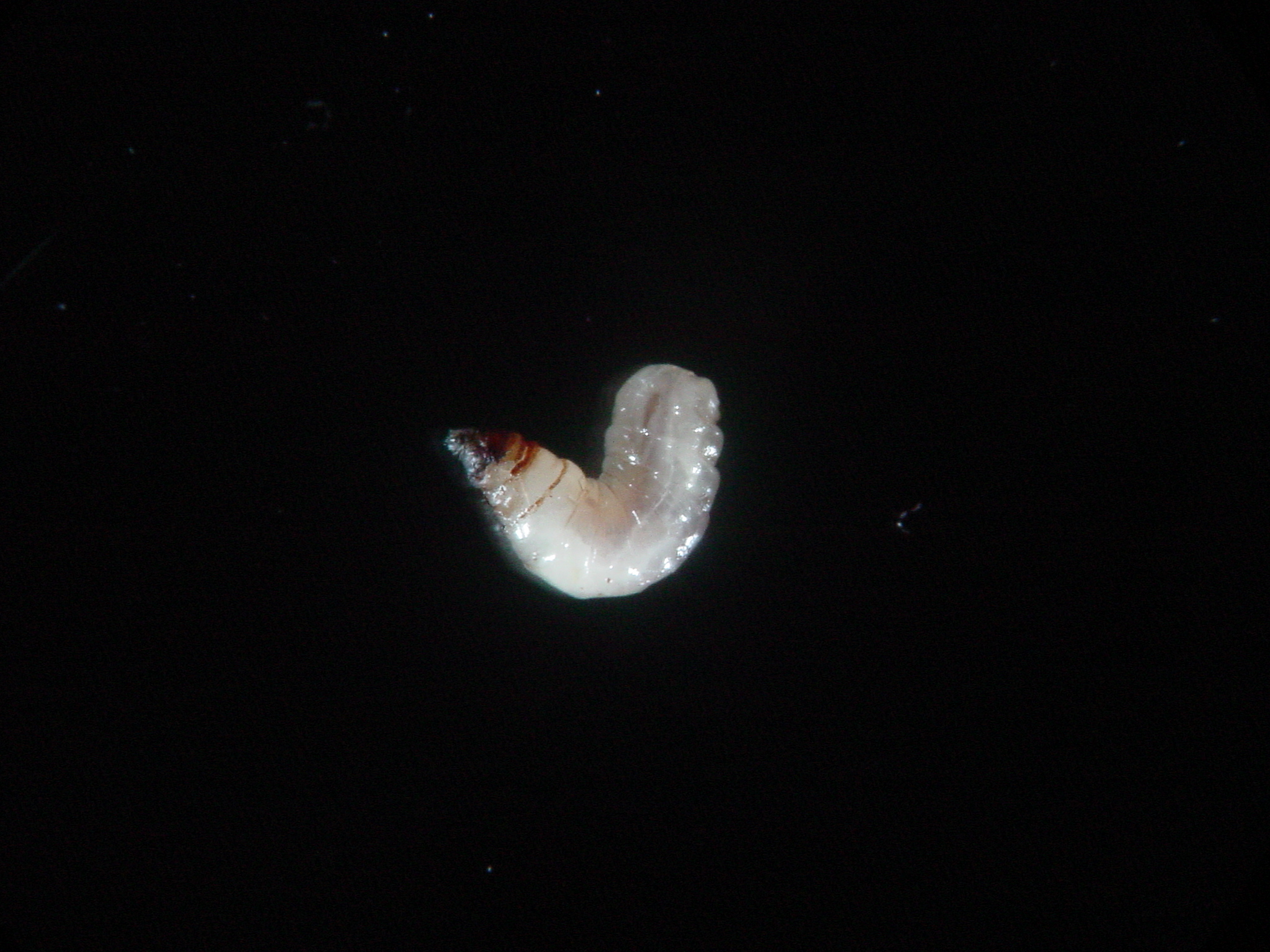

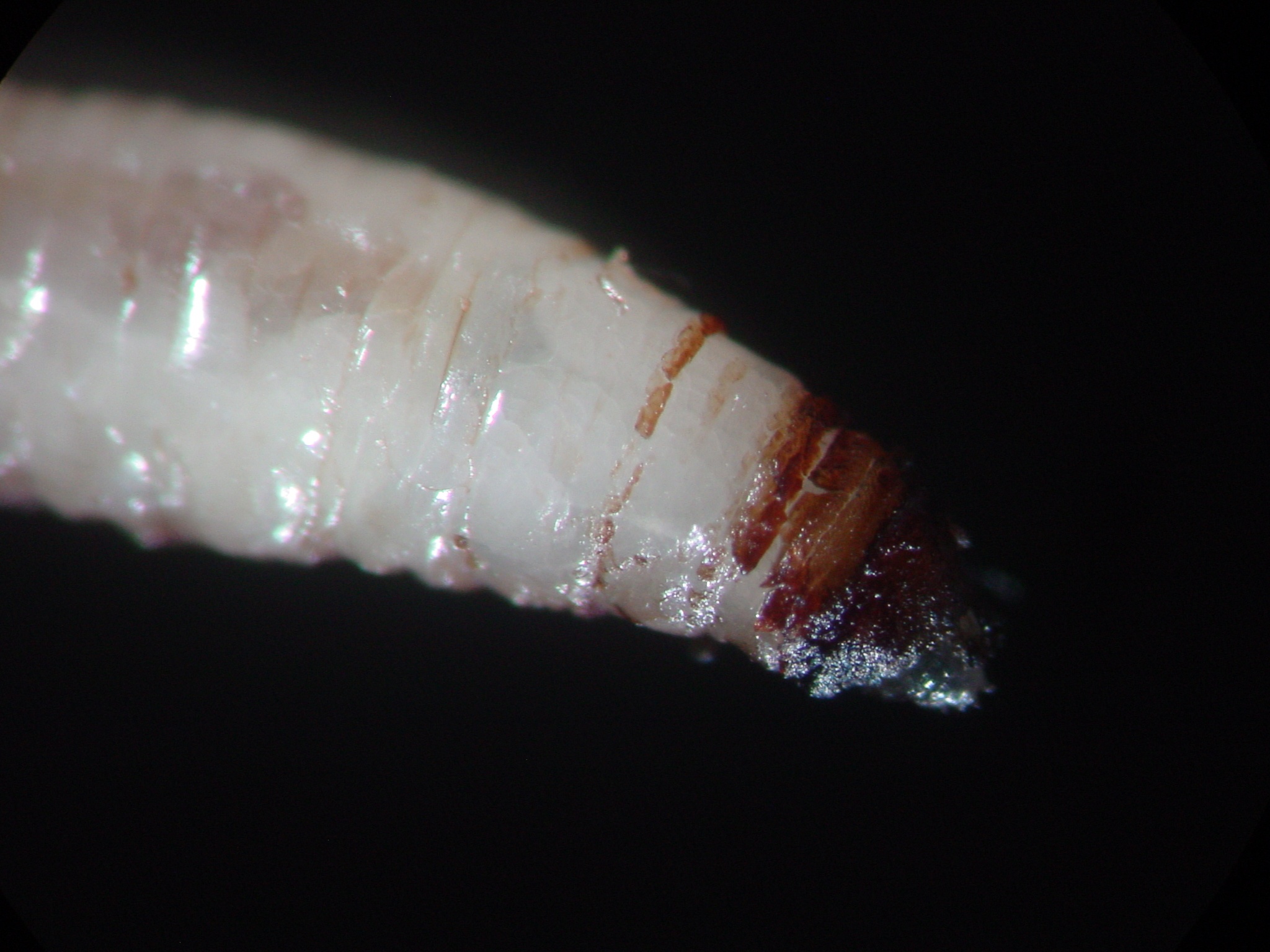

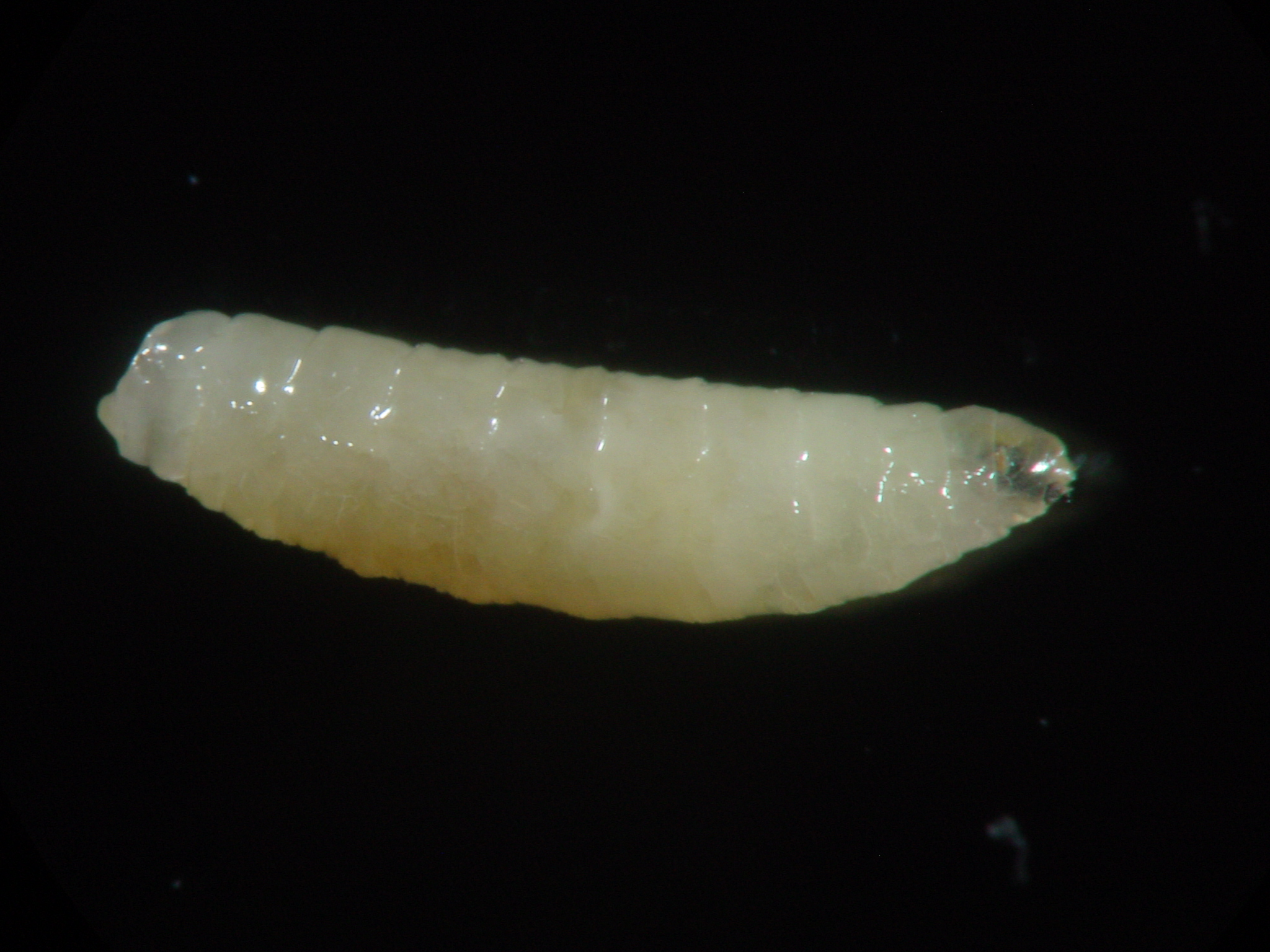

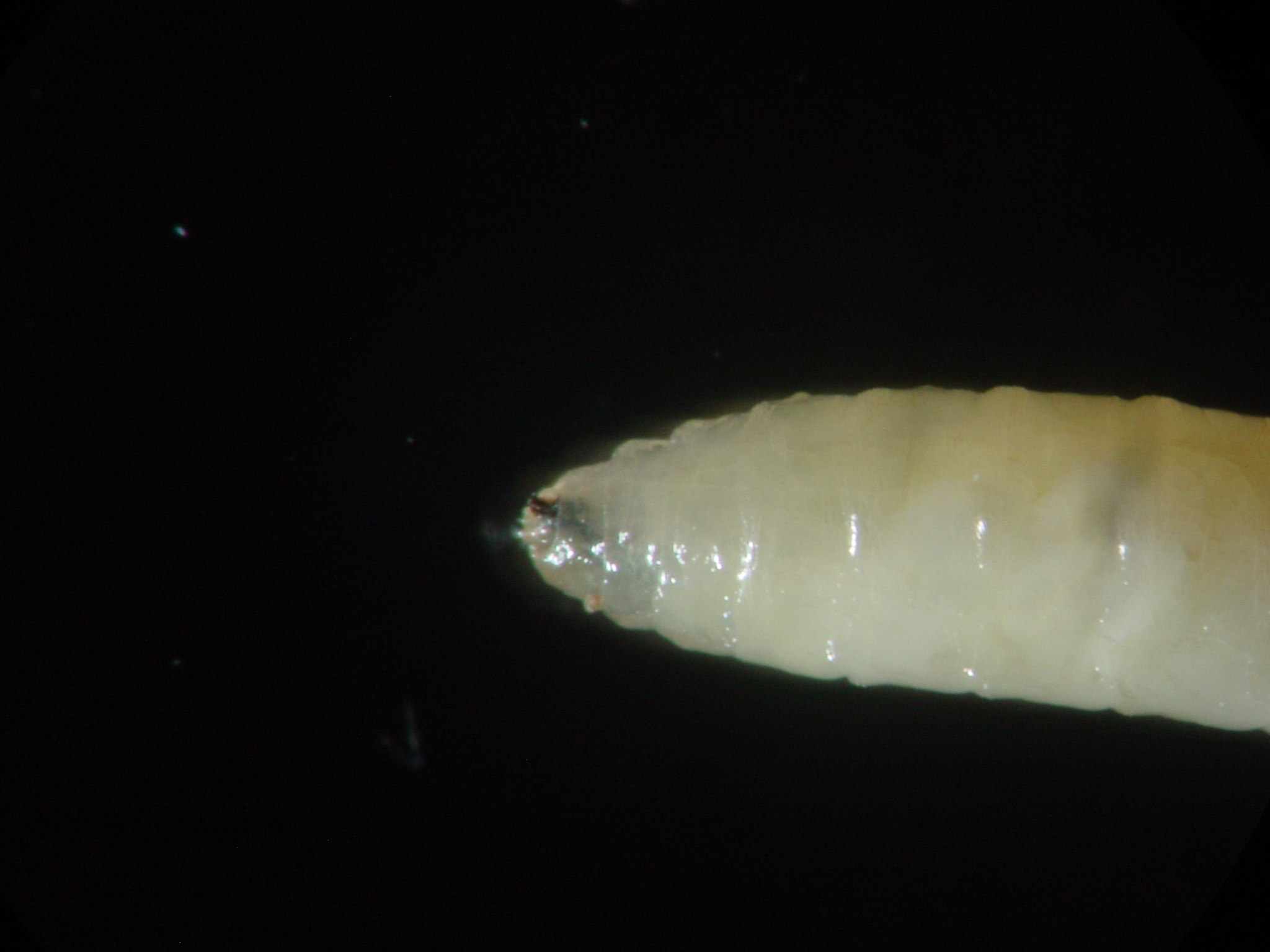

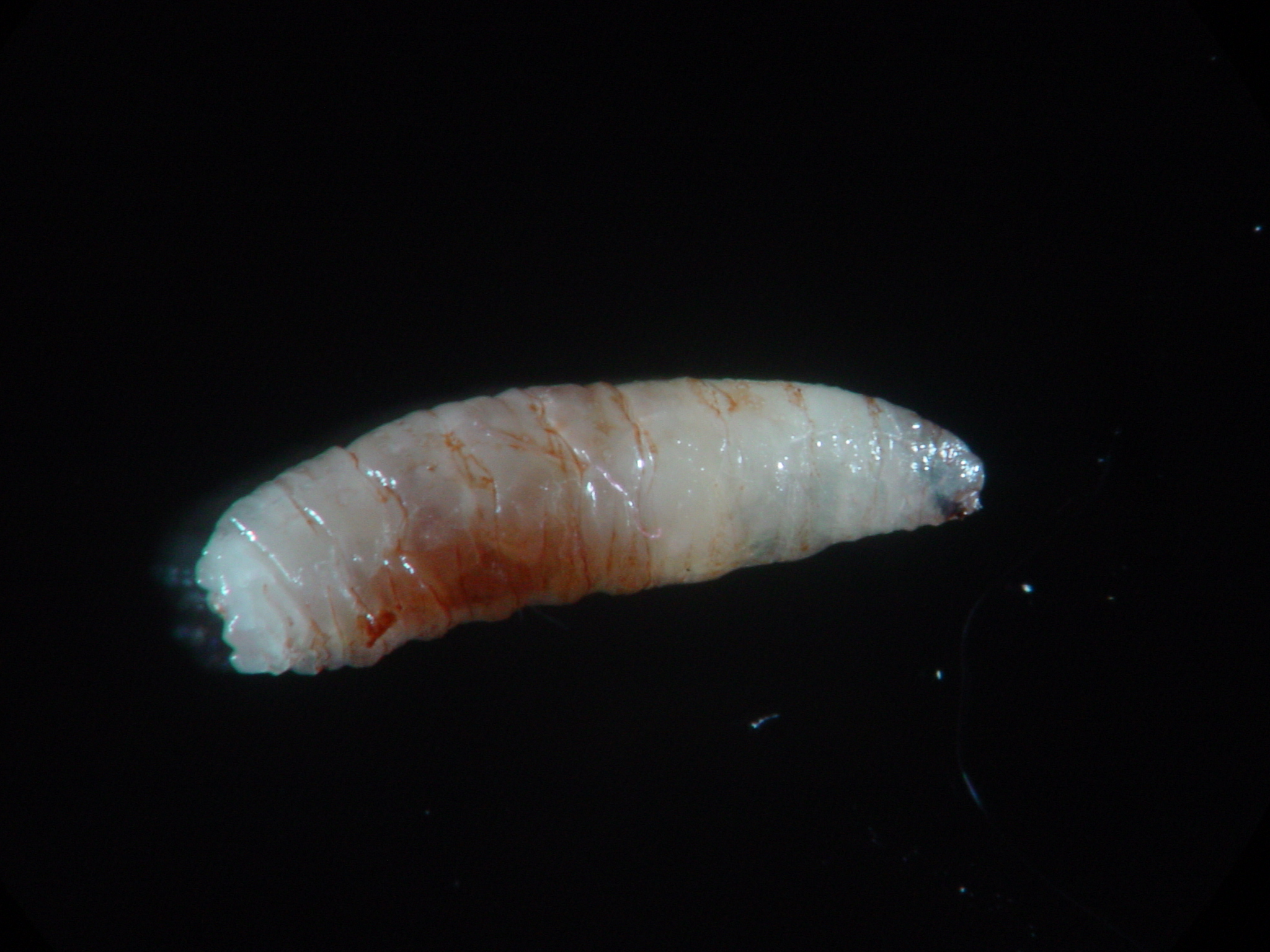

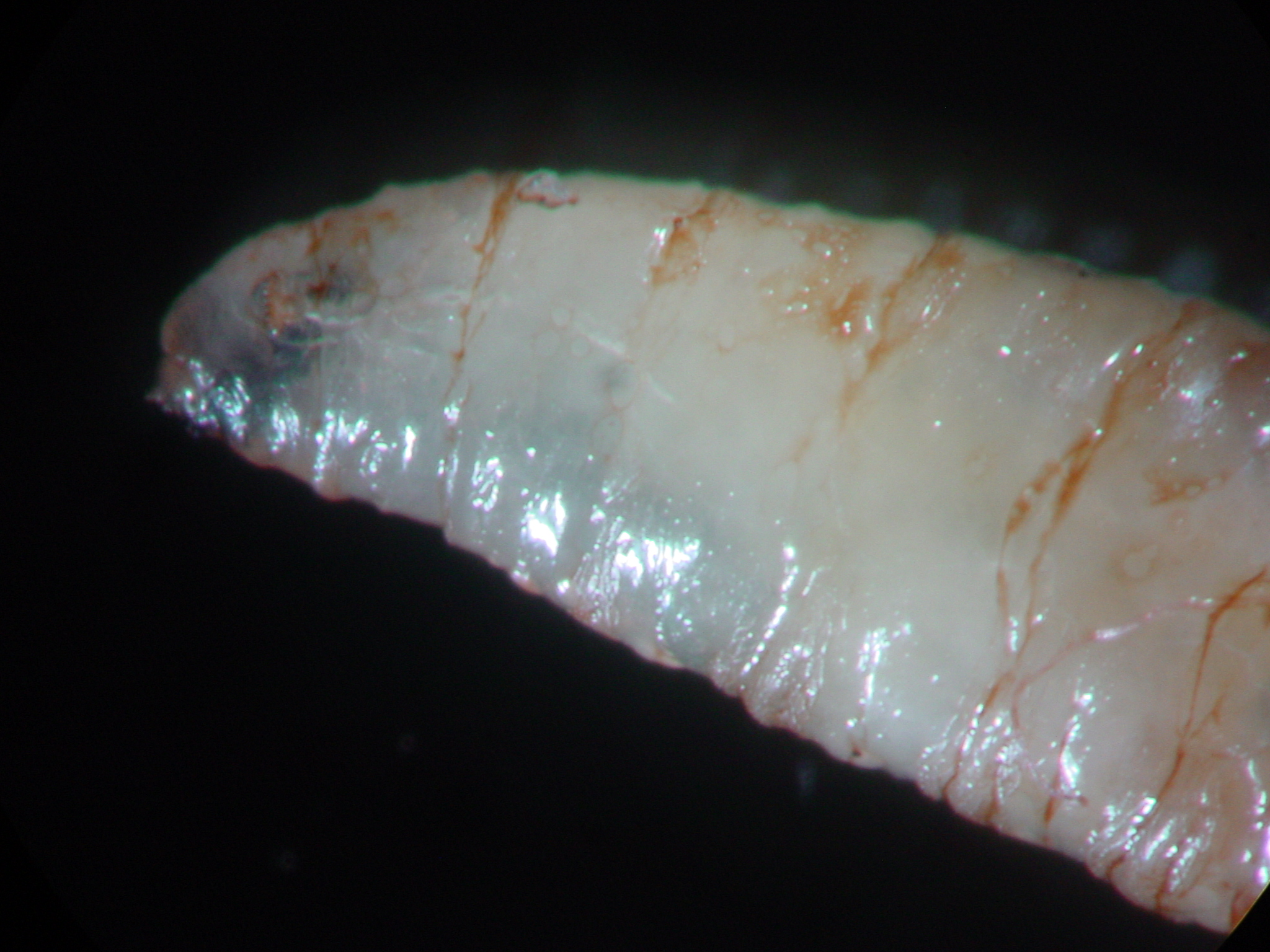


a

b

c

d

e

f

**Figure S3:** rarefaction curves of sequencing data, denoting the low number of OTUs in the samples as well as the ‘saturation’ of the curves which indicate that all the available OTU diversity in the samples was detected.

**Table S1:** Full factorial analysis examining the effects of 'treatment' (symbiotic or aposymbiotic), 'fruit weight / number of larvae' and 'olive variety' (Suri or Manzanillo) on pupal weights (ANOVA) and larval periods (survival analysis) of males and females developing in unripe or ripe olives.

**Table S2:** Linear regression analysis examining the effect of amount of resources ('fruit weight / number of larvae') on pupal weight and larval period of symbiotic and aposymbiotic males and females developing in unripe or ripe olives.
